# STAT3 but Not STAT1 Is Required for Astrocyte Differentiation

**DOI:** 10.1371/journal.pone.0086851

**Published:** 2014-01-23

**Authors:** Seulgi Hong, Mi-Ryoung Song

**Affiliations:** School of Life Sciences, Bioimaging Research Center and Cell Dynamics Research Center, Gwangju Institute of Science and Technology, Oryong-dong, Buk-gu, Gwangju, Republic of Korea; UT Southwestern Medical Center at Dallas, United States of America

## Abstract

The JAK-STAT signaling pathway has been implicated in astrocyte differentiation. Both STAT1 and STAT3 are expressed in the central nervous system and are thought to be important for glial differentiation, as mainly demonstrated *in vitro*; however direct *in vivo* evidence is missing. We investigated whether STAT1 and STAT3 are essential for astrocyte development by testing the STAT responsiveness of astrocyte progenitors. STAT3 was absent in the ventricular zone where glial progenitors are born but begins to appear at the marginal zone at E16.5. At E18.5, both phospho-STAT1 and phospho-STAT3 were present in glial fibrillary acidic protein (GFAP)-expressing white matter astrocytes. Overexpression of STAT3 by electroporation of chicks *in ovo* induced increased numbers of astrocyte progenitors in the spinal cord. Likewise, elimination of STAT3 in *Stat3* conditional knockout (cKO) mice resulted in depletion of white matter astrocytes. Interestingly, elimination of STAT1 in *Stat1* null mice did not inhibit astrocyte differentiation and deletion of *Stat1* failed to aggravate the glial defects in *Stat3* cKO mice. Measuring the activity of STAT binding elements and the *gfap* promoter in the presence of various STAT mutants revealed that transactivation depended on the activity of STAT3 not STAT1. No synergistic interaction between STAT1 and STAT3 was observed. Cortical progenitors of *Stat1* null; *Stat3* cKO mice generated astrocytes when STAT3 or the splice variant Stat3β was supplied, but not when STAT1 was introduced. Together, our results suggest that STAT3 is necessary and sufficient for astrocyte differentiation whereas STAT1 is dispensable.

## Introduction

One of the major biological roles of the JAK-STAT signaling pathway is the production of astrocytes in the nervous system. When stimulated by the gp130 cytokines, leukemia inhibitory factor (LIF), ciliary neurotrophic factor (CNTF) and cardiotrophin-1 (CT-1), cortical progenitors readily become astrocytes expressing the mature astrocyte marker glial fibrillary acidic protein (GFAP) [1]. Similarly, elimination of the corresponding receptors results in the loss of astrocytes [2], [3]. The activated gp130 receptor complexes activate JAK, which in turn phosphorylates STAT proteins. The activated phospho-STAT proteins dimerize and translocate to the nucleus where they bind to specific DNA binding motifs and turn on transcription of genes involved in glial differentiation [4].

There are multiple STAT proteins and they form either heterodimers or homodimers depending on the cellular context. For example, STAT1 heterodimerize with STAT2 or STAT3, in response to interferon signaling in the immune system [5], [6]. Similarly, STAT1 and STAT3 are expressed in the developing CNS, and mediate the cytokine-gp130 signaling that induces glial differentiation. However, it is uncertain what the respective roles of STAT1 and STAT3 are, whether they are equally potent or synergistic each other. STAT1 and STAT3 form heterodimers that bind to the *gfap* promoter, at least *in vitro* [7]. The affinity of these heterodimers could be different from the homodimers and, more importantly, their biological activity in glial differentiation has never been tested *in vivo* [8]–[10]. There is some evidence that STAT1 and STAT3 differ in their gliogenic potential. *Stat1* null mice are viable and only have minor defects in immune responses postnatally [11]. Astrocyte formation in these animals is normal, indicating that STAT1 may be dispensable for gliogenesis [12]. On the other hand, genetic elimination of *Stat3* results in severe astrogliosis defects, which suggest that STAT1 may not be as potent as STAT3 [13]–[15].

To determine whether STAT1 and STAT3 have different abilities to promote astrocyte formation *in vivo*, we compared their potency using a variety of experimental approaches. Overexpression of STAT3 induced glial markers in the neural tube, and elimination of *Stat3* inhibited astrocyte differentiation. By contrast, the absence of STAT1 did not disrupt glial differentiation nor worsen the defects in *Stat3* conditional knockout (cKO) mice. Finally, introduction of exogenous STAT3, but not of STAT1, rescued the glial defects in a genetic background lacking both STAT1 and STAT3. Taken together, our results show that STAT3 is necessary and sufficient for astrocyte differentiation and that STAT1 plays a minimal role, if any, in it.

## Methods

### Mouse Lines

The generation of *Stat1* KO, *Stat3* flox mice has been reported previously [11], [16]. *Stat1* KO; *Stat3^fl/fl^*; *Nestin::Cre* (cKO) mutant mice were obtained from crosses between a male *Stat1* KO; *Stat3^fl/+^*; *Nestin::Cre* mouse and a female *Stat1* KO; *Stat3^fl/fl^* mouse. The mice were housed in specific pathogen-free barrier facilities and used in accordance with protocols approved by the Animal Care and Ethics Committees of the Gwangju Institute of Science and Technology (GIST). The day of vaginal plug formation was designated embryonic day 0.5 (E0.5).

### Plasmid Construction

The *Stat3* CA (contains A662C, N664C mutations), *Stat3* Y705F, *Stat3* S727A, *Stat3β* and *Stat1* Y701F plasmids were generated by site-directed mutagenesis using primer pairs reported in previous studies [17]–[21].

### Primary Cortical Culture and Retroviral Infection

Primary cortical cultures were established as described previously [22]. CNTF (100 ng/ml, R&D system) was added to cells once 3 hrs after plating and the cells were harvested at 6 days *in vitro* (DIV) for further immunocytochemical analysis. For retrovirus production, the Phoenix ecotrophic retroviral packaging cell line (ATCC) and *pBMN-GFP* retroviral expression vector were used [23]. Low-titer retrovirus was applied to the cortical culture immediately after plating.

### Immunoblotting and Immunoprecipitation (IP)

Dissected spinal cords or cells were lysed and analyzed by Western blot analysis [24]. Antibodies used were: rabbit anti-STAT3 (Santa Cruz), rabbit anti-STAT1 (Santa Cruz), rabbit anti-pSTAT3 (Tyr 705) and anti-pSTAT1 (Tyr 701) (Cell Signaling Technology), mouse anti-α tubulin (Sigma), and mouse anti-GFAP (Chemicon). IP experiments were performed as described previously [25]. HEK-293T cells were transfected with *pCDNA3-myc-p300* and *pBOS-flag-Stat1* or *pBOS-flag-Stat3*. After serum starvation, CNTF (100 ng/ml) were treated. After 0.5 or 1.5 hours, cells were lysed in IP lysis buffer with protease inhibitor cocktail (Calbiochem). Lysate were immunoprecipitated with anti-FLAG M2 affinity gel (Sigma). Immunoprecipitates were analyzed by Western blot analysis using anti-Myc- (Cell Signaling Technology) and anti-FLAG M2-Peroxidase (Sigma) antibodies.

### Chick Electroporation

For long-term chick electroporation, *Stat3* and *Stat3* CA genes were subcloned into *pT2K-CAGGS* vector with *IRES-EGFP* [26]. Conditions for *in ovo* electroporation were described previously, and embryos were harvested on Day (D) 15 [27].

### Immunostaining

Mouse or chick embryos were harvested and processed for cryosection (Leica). The following antibodies were used for immunostaining: rabbit anti-STAT3 (Santa Cruz), rabbit anti-pSTAT1 (Ser727) (Cell Signaling Technology), rabbit anti-GFAP (Dako), monoclonal anti-GFAP-Cy3™ (Sigma), guinea pig anti-Olig2 (Dr. Jessell), rabbit anti-NFIA (Active Motif), rabbit or mouse anti-GFP (Invitrogen), mouse H5 (DSHB).

### In situ Hybridization

To generate riboprobes, DNA sequences for *GLAST* (nt 1053-1765 of XM425011) and *Hes5* (nt 18-1076 of AY916777) were amplified by PCR from a chick D7 whole embryo cDNA library. In situ hybridization was performed as previously described [24].

### Luciferase Assay

The *gfap* minimal promoter (GFMP) with 8 repeats of the STAT binding site (SBS8GFMP) and 2.5 kb of the rat *gfap* promoter were used [3], [28]. COS-7 cells or primary cortical cells from E16.5 brains were transfected with the reporter constructs and STAT3, STAT1 or their mutants. A β-galactosidase plasmid was co-transfected as an internal control. Cells were incubated with CNTF (100 ng/ml) for 12 hrs at 2 DIV before they were harvested. Cell lysates were assayed for luciferase (Promega) and β-galactosidase (Sigma). Data for luciferase were normalized with β-galactosidase activity.

### Statistical Analysis

Staining data are means ± SEM of more than 5 sections from at least three separate embryos. For cortical cultures and reporter assays, three independent experiments were performed in triplicate. Asterisks indicate statistically significant differences in unpaired-Student’s t-test (*p<0.05, **p<0.01, ***p<0.001). Comparisons between multiple groups were made with one-way ANOVA with Tukey’s *post hoc* multiple comparison tests.

## Results

### STAT3 is Selectively Expressed in Mature White Matter Astrocytes

**Figure 1:**
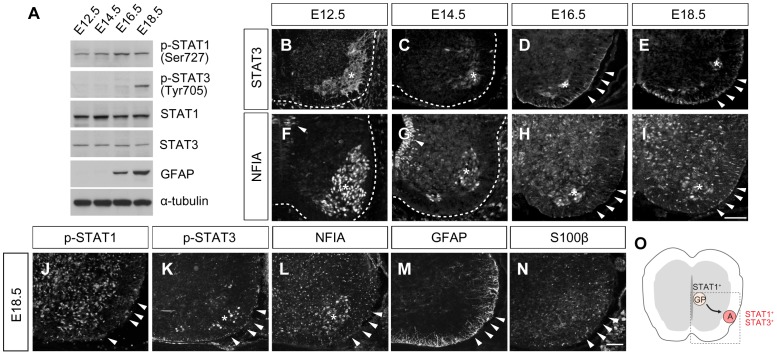
Expression of STAT and astrocyte markers in the spinal cord during CNS development. **(**A) Western blot analysis of phospho-STAT1 (Ser 727) (p-STAT1), phospho-STAT3 (Tyr 705) (p-STAT3), STAT1, STAT3 and GFAP in the mouse embryonic CNS. α-Tubulin served as loading control. (B–I) Expression of STAT3 and NFIA in the spinal cord. STAT3 is present in E16.5 and E18.5 white matter astrocytes (D, E, arrowheads). NFIA expression is seen in migrating glial progenitor cells and overlaps with STAT3 expression in the marginal zone (F–I, arrowheads). (J–N) Expression of p-STAT1, p-STAT3, NFIA, GFAP and S100β in E18.5 spinal cords. P-STAT1 and p-STAT3 are present in astrocytes at the marginal zone (J–N, arrowheads). Note that motor neurons also express STAT3 and NFIA (B–I, K, L, asterisks). (O) Diagram of STAT expression in glial progenitors (GP) and astrocytes (A) in the developing spinal cord. Scale bar: in I, 100 µm for B–I; in N, 100 µm for J–N.

To test whether STAT3 is expressed in the developing central nervous system (CNS), we first examined its expression in spinal cord lysates of E12.5, E14.5, E16.5 and E18.5 mouse embryos by Western blot analysis (Fig. 1A). We focused on astrocytes in the spinal cord since they are easy to locate during the embryonic period and we planned to examine gliogenesis in *Stat3* mutant mice, which are embryonic lethal [16]. STAT1 and phospho-STAT1 were expressed in all conditions. Interestingly, phospho-STAT3 was only found at E18.5, although STAT3 was present from E12. The appearance of phospho-STAT3 coincided approximately with the expression of the astrocyte marker GFAP at E16.5, suggesting that STAT3 might be more relevant to gliogenesis than STAT1. Next, we examined STAT3 expression in the spinal cord by immunohistochemistry. In the spinal cord, progenitors are located in the ventricular zone next to the midline and migrate laterally. In particular, white matter astrocytes spread over the mantle zone (grey matter) and reach the marginal zone (white matter) where they undergo maturation (Fig. 1O). In E12.5 and E14.5, when neurogenesis is ongoing, STAT3 expression was limited to the marginal zone and postmitotic motor neurons (Fig. 1B, C). At E16.5 and E18.5, when astrocyte differentiation begins, STAT3 immunoreactivity appeared in the marginal zone, and overlapped with that of NFIA, which are present in glial lineage cells (Fig. 1D, E, H, I) [29]. Nuclear expression of phospho-STAT1 was dense in the grey matter and also found in the white matter (Fig. 1J). By contrast, Phospho-STAT3 expression was low in the grey matter but was induced in the white matter at E18.5, coincident with expression of STAT3, NFIA and glial markers S100β and GFAP. Thus, STAT3 is selectively expressed in differentiated white matter astrocytes (Fig. 1K–N).

### Misexpression of STAT3 Induces Ectopic Glial Cells at Late Periods of Glial Development

**Figure 2:**
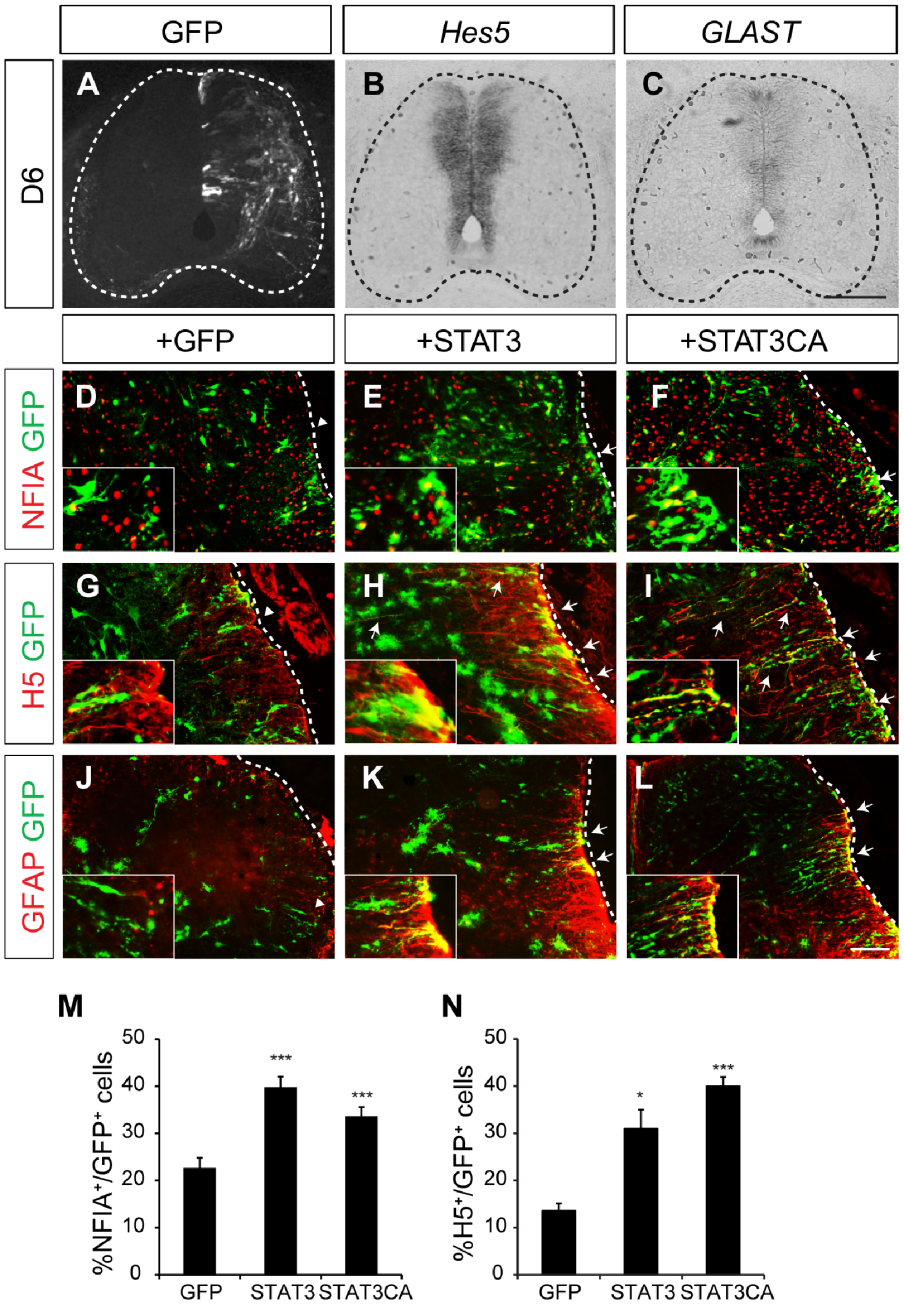
Overexpression of STAT3 in the chick spinal cord induces differentiation of astrocytes. (A–C) Expression of glial markers *Hes5* and *GLAST* in STAT3-overexpressing chick spinal cords at D6, which is not changed. (D–L) Expression of GFP, NFIA, H5 and GFAP in D15 chick spinal cords electroporated with GFP, STAT3 or STAT3CA. Insets are magnified images. GFP^+^ processes express H5 and GFAP in STAT3 or STAT3CA-electroporated groups (arrows, H, I, K, L), unlike the ones in control (arrowheads, D, G, J). Dotted lines mark the boundary of neural tube. (M, N) Quantification of NFIA and H5-expressing cells in the marginal zone. Error bars represents s.e.m. *p<0.05, ***p<0.001 vs. GFP; unpaired-Student’s t-test. Scale bars: in C, 100 µm for A–C; in L, 100 µm for D–L.

To test whether overexpression of STAT3 stimulates astrocyte formation, we misexpressed STAT3 in the chick neural tube by *in ovo* electroporation. Since gliogenesis continues in late gestation, we used a *tol2-*transposon plasmid that allows long-term stable expression of STAT3 by genomic integration [26]. On D6, when neurogenesis is still active, there was no change in the expression of the glial progenitor markers *Hes5* and *GLAST* on the electroporated side of embryos (Fig. 2A–C). Next we examined glial cells at a later period such as D15 after electroporating STAT3 or STAT3CA, a constitutively active form of STAT3. The proportion of electroporated cells that express NFIA was significantly increased on the electroporated sides (1.8-fold and 1.5-fold of the GFP control, respectively) (Fig. 2D–F, M) [29]. The numbers of glial processes in the marginal zone labeled with glia-lineage markers H5 and GFAP were also greater on the electroporated sides (2.3-fold and 3-fold of the GFP control for H5, respectively) (Fig. 2G–L, N). Together these observations indicate that ectopic expression of STAT3 stimulates the production of glia-lineage cells at late periods of glial development.

### STAT3 but not STAT1 is Required for Astrocyte Differentiation

**Figure 3:**
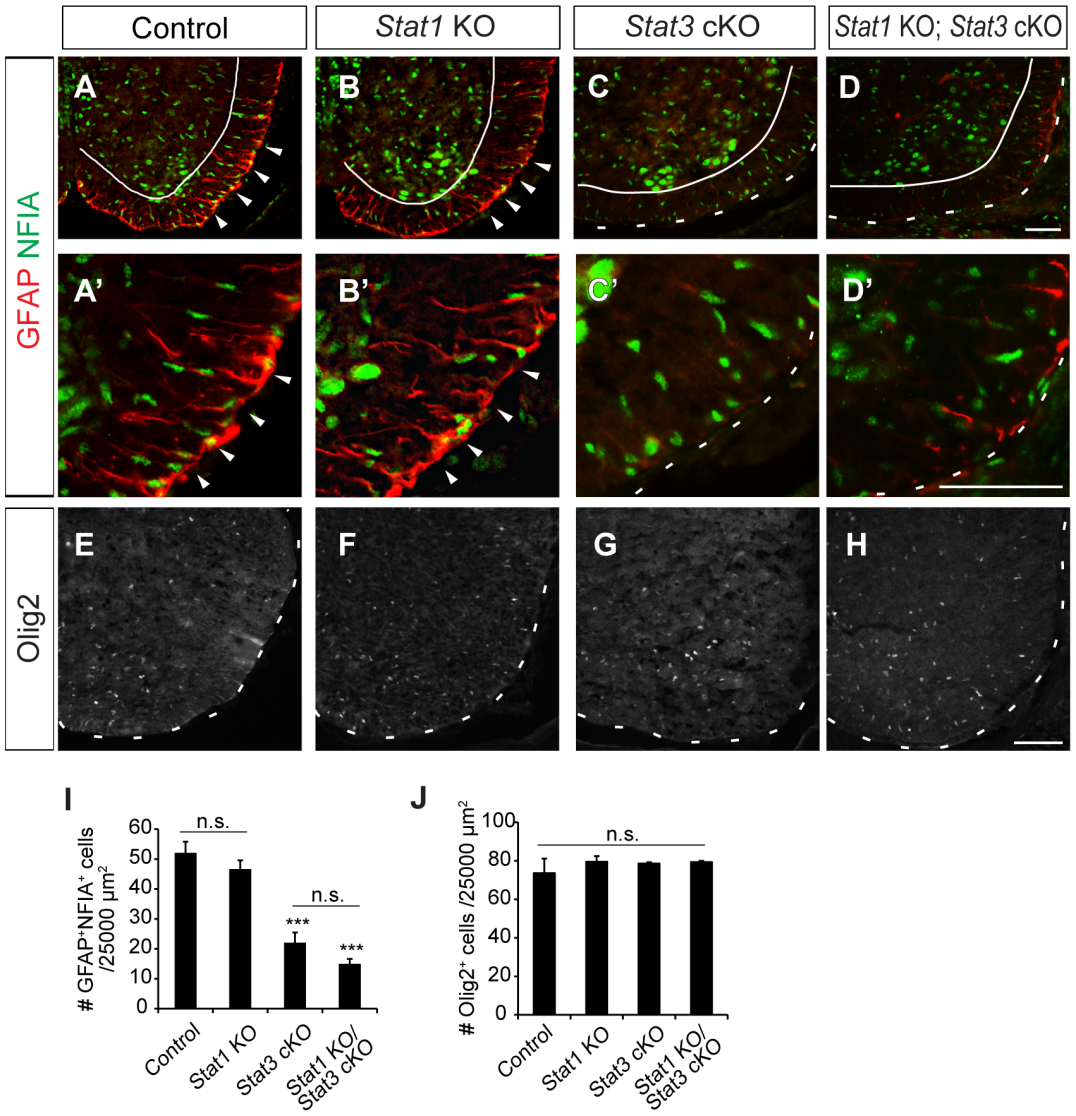
Glial development in E17.5 *Stat1* KO, *Stat3* conditional mutant mice (*Stat3^fl/fl^*; *Nestin::Cre,* cKO) and *Stat1* KO*; Stat3* cKO mice. (A–H) Expression of GFAP, NFIA, Olig2 in E17.5 spinal cords of *Stat1* KO, *Stat3* cKO and *Stat1* KO*; Stat3* cKO mutants. GFAP^+^NFIA^+^ astrocytes are present in the control and *Stat1* KO spinal cords (arrowheads, A–B’), but are greatly reduced in *Stat3* cKO and *Stat1* KO; *Stat3* cKO mice (C–D’). (I, J) Quantification of cells expressing glial markers: NFIA, GFAP and Olig2 as indicated. Error bars represents s.e.m. **p<0.01, ***p<0.001 vs. control; n.s., not significant; one-way ANOVA with *post hoc* Tukey’s multiple comparison test. Scale bars: in D, 100 µm for A–D; in D’, 100 µm for A’-D’; in H, 100 µm for E–H.

We next tested whether STAT3 is essential for gliogenesis by examining astrocyte formation in the absence of STAT3. *Stat3* knockout embryos die prior to neural tube formation [16]. Therefore, we generated neural stem cell/precursor-specific *Stat3* conditional mice (cKO) by crossing *Stat3* flox mice with *Nestin-Cre* transgenic mice. We also used *Stat1* null (KO) mice since STAT1 can form heterodimers with STAT3 [11]. We first confirmed that STAT3 protein expression was absent in the *Stat3* cKO mice but was normal in *Stat1* KO mice (Fig. S1). At E17.5, the numbers of astrocytes (GFAP^+^NFIA^+^) in *Stat1* KO mice were comparable to those in the control mice (Fig. 3A–B’, I). In contrast, the number of astrocytes in *Stat3* cKO and *Stat1* KO; *Stat3* cKO mice were reduced by 42% and 29% relative to the control mice, respectively (Fig. 3C–D’, I). The numbers of astrocytes between *Stat3* cKO and *Stat1* KO; *Stat3* cKO mice were not significantly different (Fig. 3I). Numbers of oligodendrocytes were comparable in all the animals (Fig. 3E–H, J). Thus, STAT3 is critical specifically for astrocyte formation, whereas STAT1 is dispensable.

### Glial Differentiation was Affected in *STAT3* Mutants

**Figure 4:**
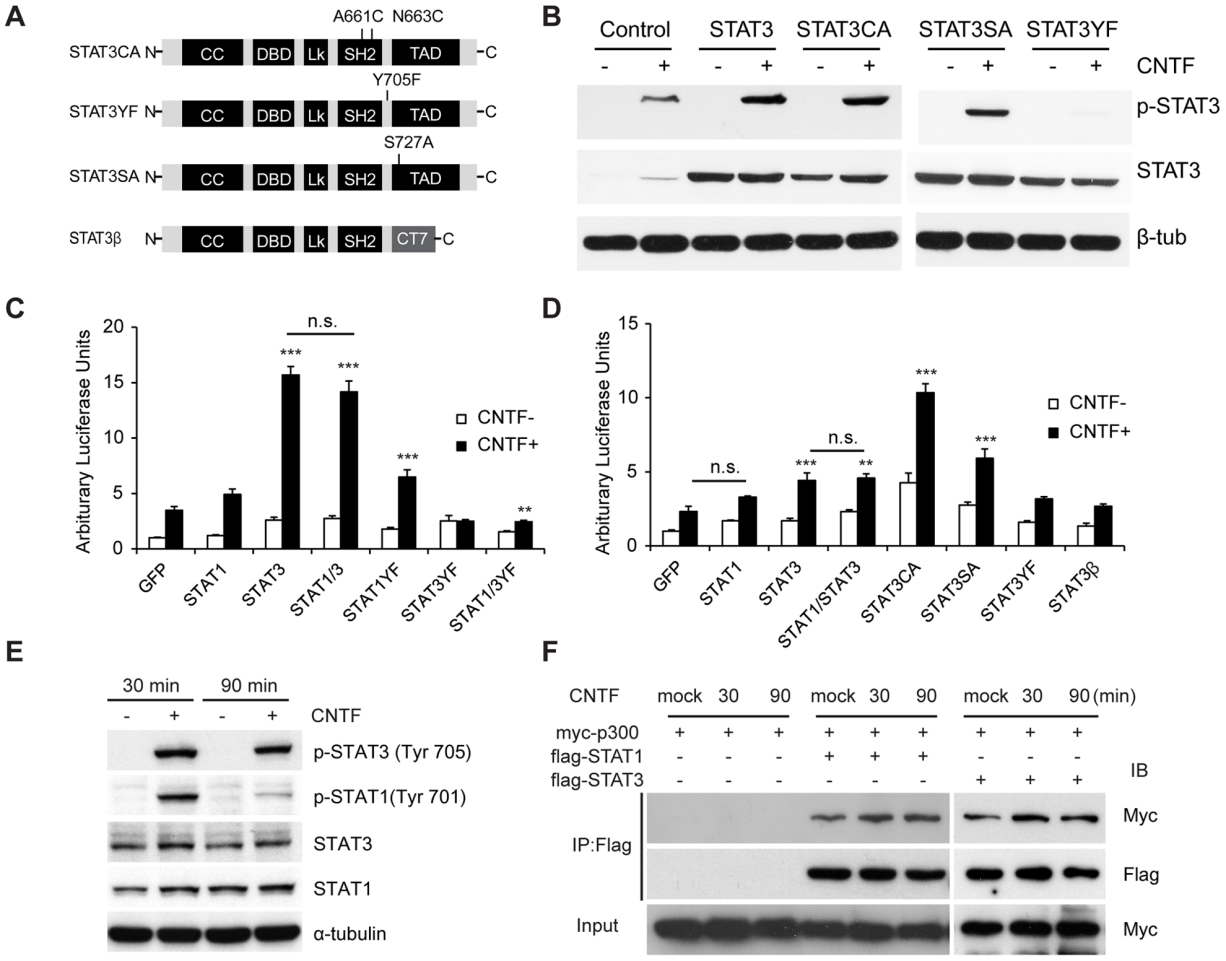
Structure-function analysis of STAT3 in astrocyte differentiation. (A) Diagrams of STAT3CA, STAT3YF, STAT3SA and STAT3β. CC, coiled-coil domain; DBD, DNA binding domain; Lk, linker domain; TAD, transcriptional activation domain. (B) Expression and phosphorylation of STAT3 wild-type or mutants assessed by Western blotting. Each construct was transfected into 293T cells, and the cells were stimulated with CNTF (100 ng/ml) for 10 min before harvesting. Expression of all the STAT mutants except STAT3YF was similar to that of wild-type, and they became phosphorylated in the presence of CNTF except STAT3YF. Expression of p-STAT3 in the control group is due to endogenous STAT3 activity. (C) Transactivation of the SBS8GFMP reporter in the presence of STAT derivatives. COS7 cell derivatives were incubated with CNTF (100 ng/ml) for 12 hrs. (D) Transactivation of the *gfap* promoter GF1L. E16.5 *Stat1* KO; *Stat3* cKO primary cortical progenitors were transfected with STATs and incubated with CNTF (100 ng/ml) for 12 hrs. (E) Assessment of p-STAT3 (Tyr 705), p-STAT1 (Tyr 701), STAT3, STAT1 and α-tubulin in the presence of CNTF (100 ng/ml) for 30 min and 90 min by Western blotting. (F) The binding between STAT and p300 in the presence of CNTF (100 ng/ml) tested by co-immunoprecipitation experiment. Error bars represents s.e.m. **p<0.01, ***p<0.001 vs. GFP with CNTF; n.s., not significant; one-way ANOVA with *post hoc* Tukey’s multiple comparison test.

To further investigate the roles of STAT1 and STAT3, we generated multiple STAT mutants and tested their activity in driving astrocyte differentiation. The mutants generated were STAT3CA, with point mutations in the SH2 domain that facilitates dimerization, STAT3YF with a point-mutation at tyrosine 705 phosphorylation site and STAT3SA with a mutation at Serine 727 phosphorylation site (Fig. 4A) [4]. STAT3β is a splice variant with different C-terminal residues [14]. When transfected into 293T cells, all the STAT3 derivatives generated normal amounts of STAT proteins (Fig. 4B). They were also normally responsive to CNTF, as determined by phospho-STAT3 levels, except for STAT3YF (Fig. 4B). Next we measured their transactivity with the SBS8GFMP reporter containing 8 consecutive copies of the STAT binding motif from the *gfap* promoter (Fig. 4C) [28]. We transfected the STAT plasmids into COS-7 cells, which express low levels of endogenous STAT3. In the GFP-transfected (control) group, a roughly 4-fold increase was observed in response to CNTF, presumably mediated by endogenous STAT. STAT3 produced strong induction of the reporter (4.5-fold compared to the GFP control with CNTF). By contrast, STAT1 induced weakly (1.4-fold compared to the GFP control with CNTF) and did not enhance the reporter activity driven by STAT3 (4.1-fold compared to the GFP control with CNTF). Conversely, STAT1YF, which is expected to block STAT1 activity, did not reduce transactivity in the controls, unlike dominant-negative STAT3YF, which abolished CNTF responsiveness. Moreover, co-transfection of STAT1YF did not increase the inhibition of transactivity by STAT3YF.

To measure the ability of STAT proteins to induce *GFAP* transcription in glial progenitors, we measured the activity of the 2.5 kb *gfap* promoter GF1L containing the STAT binding motif in E16.5 primary cortical cells. To minimize the effect of endogenous STAT proteins, we cultured primary cells from *Stat1* null; *Stat3* cKO brains. When GFP was expressed, a low level of CNTF-responsiveness of GF1L transactivity was observed, probably due to remnant STAT3 protein in *Stat3* cKO mice (Fig. 4D). When STAT1 was transfected into *Stat1* null; *Stat3* cKO cells, reporter activity was similar to the one in the control group. By contrast, introduction of STAT3 alone or co-transfection of STAT1 and STAT3 substantially increased transactivity (1.9-fold and 2.0-fold, respectively, compared to the GFP control with CNTF). STAT3CA and STAT3SA were also effective, while STAT3YF or STAT3β was not (Fig. 4D). Thus, STAT3 but not STAT1 transactivates the *gfap* promoter.

Distinct responses of STAT1 and STAT3 to CNTF prompted us to reason that they may deliver the cytokine signaling differently. Thus, we compared the activity of STAT proteins in various conditions with cytokines. E16.5 primary cortical cells were treated with brief (30 min) or prolonged (90 min) stimulation of CNTF. Phosphorylation of STAT3 occurred within 30 min and was maintained until 90 min in response to CNTF, assessed by Western blotting (Fig. 4E). By contrast, phospho-STAT1 (tyrosine 701) was detected in the presence of CNTF at 30 min but its level dropped at 90 min after the stimulus. Our results suggest that STAT3 signaling persists longer than STAT1 in response to CNTF and could be more potent.

During glial differentiation, recruitment of coactivator p300 by STAT3 on *gfap* promoter is critical for its transcription [3]. To test whether STAT1 also binds to p300, we conducted co-immunoprecipitation experiment between STAT proteins and p300 (Fig. 4F). Flag-STAT3 (or Flag-STAT1) and Myc-p300 were co-expressed in 293T cells and cell lysates were immunoprecipitated with anti-FLAG antibody. The interaction between STAT3 and p300 increased after 30 min and 90 min of CNTF treatment. More binding of Flag-STAT1 to Myc-p300 was also observed in response to CNTF. Thus, the recruitment of p300 by STAT1 appears to be comparable to the one by STAT3.

**Figure 5:**
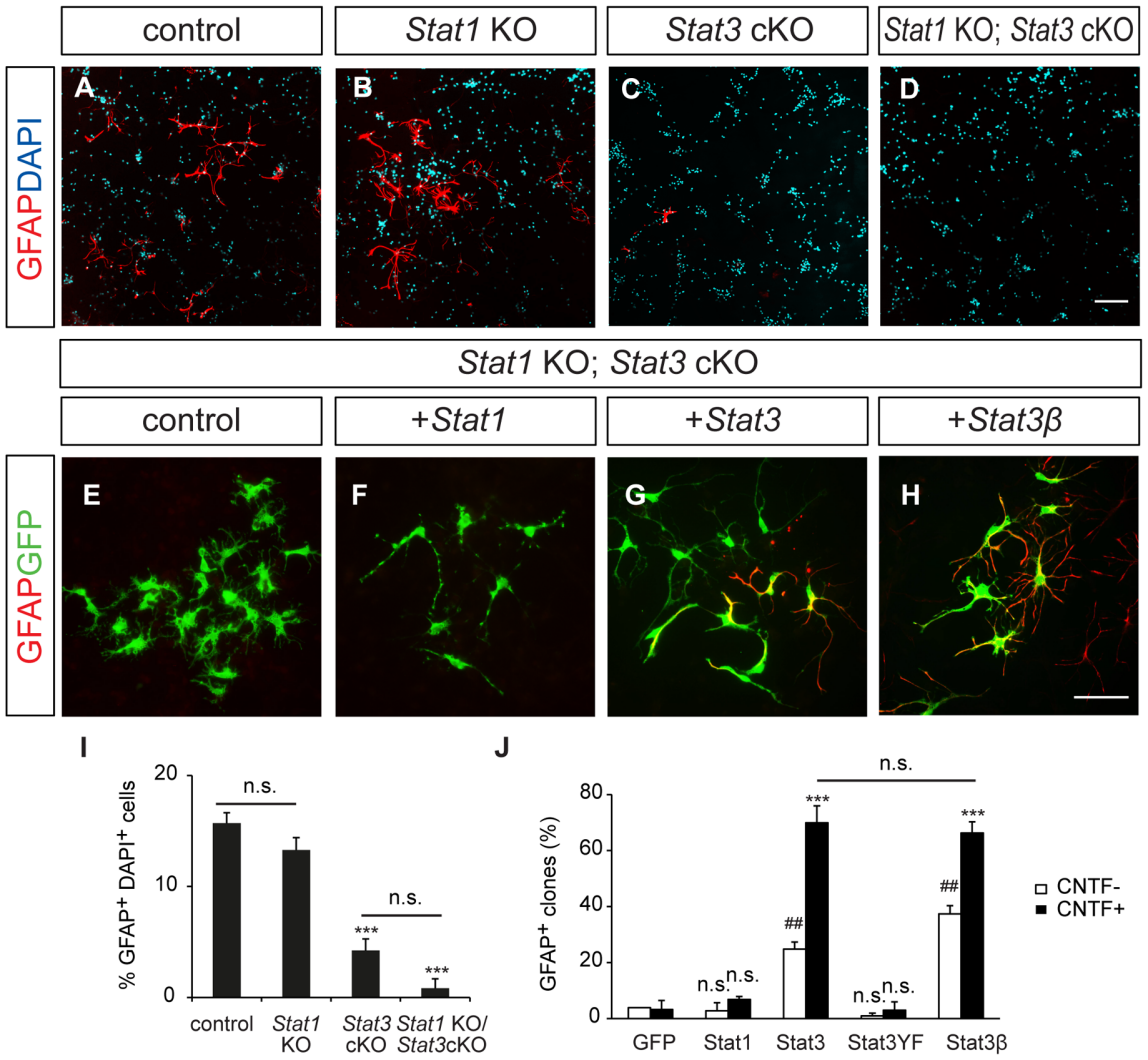
Astrocyte differentiation in E16.5 *Stat1* KO*; Stat3* cKO mutant cells in response to exogenous STAT1 and STAT3. (A–D) E16.5 primary cortical cultures of littermate controls, and *Stat1* KO, *Stat3* cKO and *Stat1* KO; *Stat3* cKO mice. Cells were grown in the presence of CNTF for 6 days and immunostained for GFAP (red). (E–H) Cortical cells from E16.5 *Stat1* KO; *Stat3* cKO mice were infected with *GFP, Stat1*, *Stat3* and *Stat3β* retrovirus and grown in the presence of CNTF for 6 days. (I) % GFAP-labeled cells among DAPI-labeled cells. (J) Quantification of GFAP-expressing cells in each condition. Error bars represents s.e.m. ***p<0.001 vs. GFP with CNTF; ##*p<*0.01 vs. GFP with no CNTF; n.s., not significant; one-way ANOVA with *post hoc* Tukey’s multiple comparison test. Scale bars: in D, 100 µm for A–D; in H, 100 µm for E–H.

To test whether the STAT proteins are required for glial differentiation, we isolated glial progenitors from E16.5 *Stat* mutant brains and tested their ability to generate astrocytes *in vitro*. Cells were grown in the presence of CNTF to stimulate astrocyte differentiation and harvested at 6 DIV (Fig. 5A–D). About 15.7% and 13.3% of cells expressed GFAP in the control group and *Stat1* KO group, respectively (Fig. 5I). In contrast, very low GFAP expression was found in cells from *Stat3* cKO (4.2%) or *Stat1* KO; *Stat3* cKO mice (0.8%), indicating that astrocytes cannot differentiate without STAT3.

Next, we compared the activities of STAT1 and STAT3 in inducing astrocytes in cortical progenitors. When we misexpressed the STAT proteins in wild-type cortical cultures, astrocyte differentiation was not affected, probably because endogenous levels of STAT were sufficient (data not shown). Therefore we examined the activities of exogenous STATs in *Stat1* KO; *Stat3* cKO cells. Primary E16.5 cortical cultures from *Stat1* KO; *Stat3* cKO mice were infected with *GFP, Stat1* or *Stat3* retroviruses and grown in the presence of CNTF for 6 DIVs. Almost no GFAP expression was found in the cells receiving GFP virus (3.9% in the control, 3.2% in CNTF-treated group) (Fig. 5E, J). STAT1 retrovirus induced virtually no GFAP expression either (2.8% in the control, 6.8% in CNTF-treated group) (Fig. 5F, J). GFAP expression was greatly enhanced by *Stat3* retrovirus (25% of cells in the control, 70% in the CNTF-treated group) (Fig. 5G, J). The induction of astrocytes by *Stat3* virus without CNTF treatment may be explained by the presence of endogenous CNTF. When STAT3YF was introduced, few glial progenitors became astrocytes (1% in the control, 3% in the CNTF-treated group) (Fig. 5J). On the other hand, STAT3β gave rise to as many astrocytes (37% in the control, 66% in CNTF-treated group) as wild-type STAT3α (Fig. 5H, J). Thus to summarize: tyrosine 705 of STAT3 is critical for STAT3 stimulation of astrocyte differentiation; STAT3β is as potent as STAT3α, while STAT1 is essentially ineffective.

## Discussion

Cytokine signaling has been suggested to be important for astrocyte differentiation but the contribution of downstream signaling components is unclear due to cross-talk between them and other signaling pathways. For a long time it has been believed that both STAT1 and STAT3 activate the relevant cytokine signaling and promote gliogenesis. In the present study, we tested whether STAT1 and STAT3 are equally important for glial differentiation, using three approaches, 1) gain-of-function experiments overexpressing STAT proteins, 2) loss-of-function studies using mouse genetic models that lack STAT1 and/or STAT3, and 3) rescue experiments introducing exogenous STAT proteins into cells that lack *Stat1* and/or *Stat3*. Overexpression of STAT3 resulted in increased numbers of glial progenitors, and removal of *Stat3* led to a severe loss of astrocytes. Unexpectedly, the absence of *Stat1* did not affect astrocyte formation nor did it aggravate the glial defects in *Stat3* conditional mutant mice. Furthermore, introduction of STAT3 but not STAT1 was able to rescue the glial defects in cells lacking endogenous *Stat3*. All these findings suggest that STAT3 is critical for maturation of astrocytes, while its paralogue STAT1 is not.

### Dispensable Roles of STAT1 in Astrocytes

Gliogenesis is mainly mediated by the LIF, CNTF and CT-1 cytokines and their co-receptor, the gp130 receptor, which utilizes the JAK-STAT signaling pathway. The addition of cytokines to cell cultures, and genetic elimination of their receptors, has demonstrated that they are important for glial differentiation [2], [3], [13]. Likewise, their downstream signaling components in the JAK/STAT pathway are intimately involved in astrocyte formation. Downregulation of JAK2 inhibited activation of STAT and transcription of *GFAP* [30], while removal of STAT3 resulted in a severe reduction in numbers of astrocytes [13]–[15]. The role of STAT3 in glial differentiation has been well-characterized using the *gfap* promoter, which STAT3 binds and transactivates [31]. Detailed promoter analysis has mapped the STAT3 binding site within the *gfap* promoter that is critical for transcription. However, the role, if any, of STAT1 in these contexts is not understood [18].

STAT1 has an important role in the immune system as demonstrated by the severe immunological defects in *Stat1* null mice [4], [11]. In the postnatal CNS, STAT1 mediates inflammatory responses in the injured brain but its role during development is still unclear. It is present in the CNS during gliogenesis, and can be phosphorylated by the cytokines CNTF and LIF [18]. *In vitro* gel shift assays have demonstrated that STAT1 binds to the STAT binding element in the *gfap* promoter in response to CNTF, and heterodimer formation between STAT1 and STAT3 has been proven *in vitro* [5], [9], [32]. Although these reports suggest that STAT1 may play a role in glial differentiation, we have shown here that STAT1 is not essential and cannot replace STAT3. Our reporter assays showed that STAT1 barely activates the *gfap* promoter, and transfection of STAT1 did not enhance promoter activity driven by STAT3. Also, *Stat1* null mice are viable and have no obvious astrocyte defects [11]. Furthermore *Stat1* null cells phosphorylate STAT3 normally in response to CNTF and LIF, and produce mature astrocytes *in vitro*, and the introduction of STAT1 into *Stat1* null; *Stat3* cKO cells fails to reverse the glial defects. It is notable that STAT1 and STAT3 respond differently to CNTF in cortical cells: phospho-STAT3 lasted longer than phospho-STAT1 in the presence of CNTF. This, however, did not change the binding ability of STAT1 to interact with p300, indicating that alternative mechanisms may explain the discrepancy between STAT1 and STAT3. For instance, SH2 domains of STAT may distinguish between STAT1 and STAT3 as demonstrated by a domain swapping study [33]. Although detailed signaling mechanisms need to be characterized, it is tempting to speculate that transient activation of STAT1 by CNTF is neither necessary nor sufficient for astrocyte differentiation.

What then might be the role of STAT1 in gliosis? One possibility is that it is involved in fine-tuning STAT3 activity in glial progenitors by forming a heterodimer with STAT3. In cells of the immune systems, STAT1 forms heterodimers with STAT3 that squelch the STAT3 homodimers available for transcription, and as a result antagonizes STAT3 activity [10]. Alternatively, the heterodimers and homodimers may have distinct DNA binding affinities for different target genes, as demonstrated by the example of STAT3/STAT5 heterodimers, which bind to the cis-inducible element (SIE) in response to M-CSF whereas STAT3 and STAT5 homodimers do not [6], [34]. If the same were true in astrocytes, the absence of STAT1 might enhance or speed up the glial differentiation process. However this was not evident in the *Stat1* null mice, indicating that any fine tuning of STAT3 activity by STAT1 must be very subtle or context-dependent. Second, STAT1 may have a different function from STAT3 in astrocytes, activated by distinct ligands. Not all cytokines activate STAT1 and STAT3 equally [8], [35]. We show that the gp130 receptor cytokine CNTF activates STAT3 longer than STAT1, which may explain why STAT3 is far more efficient in glial differentiation. Likewise, interferons exclusively activate STAT1. In fact, interferon-γ is present during gliogenesis and directs oligodendrocyte progenitors to produce astrocytes [36]. Thus, it is possible that STAT1-specific signals promote glial differentiation or serve other functions in developing astrocytes.

### The Late Role of STAT3 in Astrocyte Differentiation

The relationship between STAT and astrocyte development has been reported by previous studies [7], [37], [38]. In embryonic rat hippocampal stem cells, CNTF triggered the differentiation of cortical precursors into astrocytes, as indicated by the expression of GFAP [7], [39]. These findings provide strong evidence that STAT proteins regulate astrocyte differentiation, consistent with our results showing co-localization of STAT with GFAP in the marginal zone of the spinal cord. In STAT3-overexpressed chick spinal cords, however, STAT3 failed to induce expression of early glial markers such as *Hes5* and *GLAST*. There are two possible explanations for these results. First, STAT3 is absent in the ventricular zone and only begins to appear in the intermediate zone and marginal zone of the spinal cord, indicating that STAT3 is less likely to play a role in glial progenitors located in the ventricular zone [40]. Second, epigenetic mechanisms may prevent STAT3 from inducing astrocyte specification in the early stage of astrocyte development, when the STAT binding site of *gfap* promoter is highly methylated to block transcription [41], [42]. In a previous study, early neuroepithelial cells failed to exhibit LIF-induced GFAP expression but a forced DNA demethylation allow them to do so [42]. In other studies, overexpression of NFI transcription factors resulted in an induction of *GLAST*, an early astrocyte precursor marker as well as demethylation of astrocyte-specific genes [43]. These findings suggest that epigenetic mechanisms gate the access of gliogenic nuclear complex to prevent the premature induction of astrocyte differentiation [44]. Therefore, we speculated that, although STAT3 has an activity to induce terminal differentiation of astrocytes when ectopically introduced in earlier progenitors, premature differentiation by STAT3 could be prevented by alternative mechanisms including epigenetic ones. Together, due to the spatiotemporal expression of STAT3 and epigenetic mechanisms, STAT3 mainly regulates the terminal differentiation of astrocytes.

### Structure-function Relationships of STAT Proteins in Glial Differentiation

STAT proteins undergo post-translational modifications that are critical for their activity. In particular, phosphorylation of tyrosine is absolutely required and phosphorylation of serine at the C-terminus modulates transactivity [4]. In this study, we assessed the ability of various STAT3 mutants to promote glial differentiation. STAT3YF was completely unable to activate the *gfap* promoter and failed to stimulate astrocyte formation. STAT3SA had similar potency to wild-type STAT3, indicating that the serine 727 residue is not critical. STAT3CA had elevated *GFAP* transactivity, even in the absence of ligands, and induced ectopic astrocyte-lineage cells when introduced into the neural tube, suggesting that dimerization of STAT3 is important for STAT3 activity. Interestingly, a splice variant, STAT3β that lacks the transactivation domain, was not effective in activating the *gfap* promoter or the STAT binding element but was as potent as STAT3α in inducing astrocyte formation in a STAT-null background. These are reminiscent of the observation that STAT3β is less active than STAT3α and may act as dominant negative but can replace STAT3α in immune cells when the latter is absent [14]. Similarly, in mice specifically lacking the alpha form of STAT3 but with intact STAT3β, astrocytes are present though at low levels [45]. The activity of STAT3β could be delivered by co-activators such as CBP or p300 that are recruited to *GFAP* transcription by STAT3 proteins [45], [46]. Taken together, our findings suggest that STAT3β may be as potent in glial differentiation as STAT3α, in some contexts.

## Supporting Information

Figure S1 **Absence of STAT3 protein expression in** ***Stat3*** **cKO mice.** STAT3 immunoreactivity was found in E18.5 wild-type and *Stat1* KO white matter spinal cords but was absent in *Stat3* cKO.
(TIF)
