# Supplementary figures and images for: STAT3 but Not STAT1 Is Required for Astrocyte Differentiation
Source: PLoS One. 2014 Jan 23;9(1):e86851. doi: 10.1371/journal.pone.0086851 (PMC3900679; doi:10.1371/journal.pone.0086851)

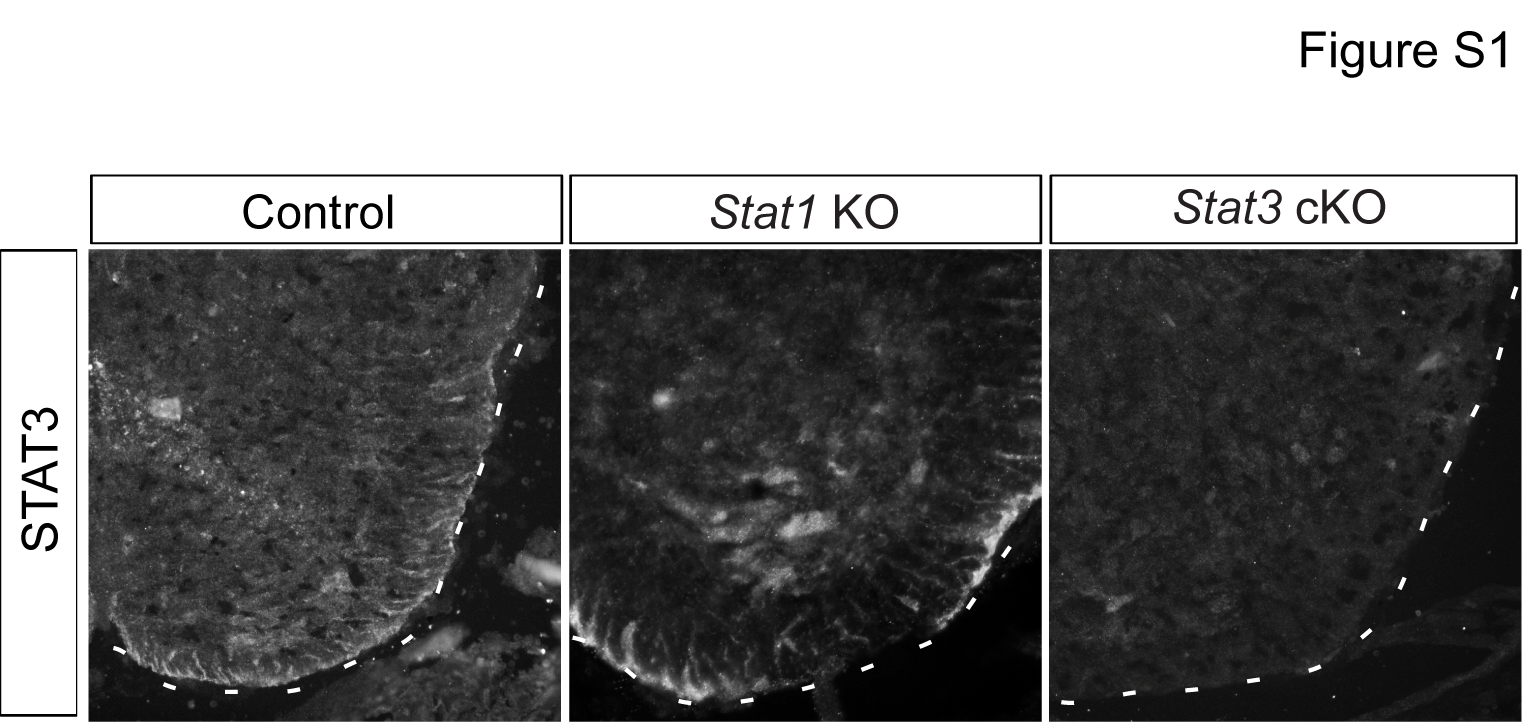

Supplement: Figure S1 — Absence of STAT3 protein expression in Stat3 cKO mice. STAT3 immunoreactivity was found in E18.5 wild-type and Stat1 KO white matter spinal cords but was absent in Stat3 cKO. (TIF) [file pone.0086851.s001.tif]
